# Dual sensitivity enhancement in gold nanoparticle‐based lateral flow immunoassay for visual detection of carcinoembryonic antigen

**DOI:** 10.1002/ansa.202000023

**Published:** 2020-06-23

**Authors:** Tohid Mahmoudi, Behnaz Shirdel, Behzad Mansoori, Behzad Baradaran

**Affiliations:** ^1^ Immunology Research Center Tabriz University of Medical Sciences Tabriz Iran; ^2^ Student Research Committee Tabriz University of Medical Sciences Tabriz Iran; ^3^ Department of Cancer and Inflammation Research Institute of Molecular Medicine University of Southern Denmark Odense Denmark

**Keywords:** carcinoembryonic antigen, gold enhancement, lateral flow Immunoassay, oriented immobilization, protein G

## Abstract

In this work, we presented the development of cost‐effective dual sensitivity enhancement in gold nanoparticle‐based lateral flow test strip for detection of carcinoembryonic antigen. On the one hand, we employed protein G as a host matrix for oriented immobilization of antibodies within the nitrocellulose membrane. On the other hand, we utilized gold enhancement approach to visualize the final signals effectively. Primary examinations revealed that the smaller sized nanoparticles have greater signal enhancement compared to bigger ones. So, mono‐dispersed gold nanoparticles with average diameters of 11.40 ± 1.40 nm were utilized as tags. The measurement of fluorescent intensity of FITC‐tagged secondary antibody attached to the polyclonal antibody, in the presence/absence of protein G as a host matrix on microplate wells, showed the successful oriented immobilization of antibodies via the host matrix. The FESEM images confirmed the attachment and growth of nanoparticles within the porous nitrocellulose membrane, after gold enhancement. Finally, under the optimized conditions, the developed strip could quantify the standard values of target within 2‐50 ng/mL range with a limit of detection of 0.35 ng/mL. This strategy enabled the reduction of antibody consumption from a conventional amount of 0.6 µg/strip down to 0.012 µg/strip. The serum samples containing carcinoembryonic antigen were also successfully analyzed by the developed strip with a visual detection limit of 10 ng/mL, which confirms favorable characteristics of the developed test strip for point‐of‐care applications.

## Graphical abstract

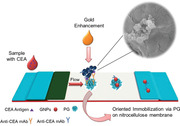

## 1 INTRODUCTION

Lateral flow immunoassays (LFIA) are point‐of‐care test strip devices with many interesting features including easy to use operation, being cheap, benefiting considerable sensitivity and specificity, rapidness, and robustness.^1^ Invented in the late 1980s, primarily the lateral flow test strips (LFTS) showed considerable attention for qualitative assays especially as pregnancy tests; nevertheless, recently the technology has been revolutionized and employed for quantification of widespread of analytes.^2^ Among them, the detection and quantification of cancer biomarkers are highly required since it provides a way for early detection, diagnosis, prognosis, checking of recurrence, and even evaluation of response to treatment.^3^ The carcinoembryonic antigen (CEA) is normally presented at very low levels in the blood but it may be elevated with certain types of cancer. Its normal range is below 5 ng/mL for healthy non‐smokers and below 10 ng/mL for smokers; however, in cancerous patients, its value in the blood usually exceeds the threshold value so it has been considered as a cancer biomarker.^4^, ^5^

Although many customary techniques, such as ELISA, are available for quantification of several analytes, the LFTS benefit from low‐costs, ease of use, rapid response, easy for on‐site decision making, and all‐in‐one assembled devices for the whole testing. However, these devices suffer from low sensitivity, the poor limit of detection, and just qualitative or semi‐quantitative results.^6^, ^7^ Due to the trace levels of biomarkers, the application of signal amplification routes is necessary for the development of sensitive test strips. The nanoparticles offer solutions for this issue since one major merit of using nanomaterials is that one can control and tailor their properties in a very predictable manner to meet the needs of specific applications.^8^ For conventional gold nanoparticle‐based LFTS, the amplification approaches include: (a) nanoparticle accumulation, (b) nanoparticle growth, (c) enzyme‐mediated color production, and (d) manipulation of the flow path to satisfy the optimum criteria for bio‐conjugation events.^9^ The second approach benefits from simplicity, considerable improvement in sensitivity, and is more interesting. Thanks to its attractive features, some studies have been recently reported for employing gold enhancement to boost the sensitivity in detection of potato virus X,^10^ *Salmonella enteritidis*,^11^ human chorionic gonadotropin,^12^ Atrazine,^13^ Avian influenza virus, and Newcastle disease virus.^14^ On the other hand, the utilization of host matrices for oriented immobilization of antibodies (Abs) is an attractive approach to retain the bioactivity of Abs within the porous nitrocellulose membrane and hence reduce their consumption, and at the same time enhance the sensitivity.^15^ Recently, the amphiphilic hydrophobin protein was introduced to modify the test line to effectively retain the Abs’ bioactivity for sensitive detection of prostate‐specific antigen.^16^ In another study, specific binding of the Fc region of an antibody with streptococcal protein G on the surface of polystyrene microspheres was utilized for quantification of cardiac troponin I.^17^ Protein G (PG), from group G Streptococci, represents a more versatile IgG‐binding reagent but it possesses a serum albumin binding site. Its recombinant form, which is genetically engineered to possess IgG‐binding activity, is commercially available and is a superior alternative to native form.^[^ ^18^ ^]^ The PG has been used as a recognition binder on magnetic nanoparticles to capture anti‐zearalenone IgG in magnetic nanobeads‐based fluoro‐immunoassays for zearalenone detection.^19^ In the same way, latex microspheres labeled with PG was used as reporting tag in LFTS for brucellosis screening.^20^ Nevertheless, its utilization as a host matrix for the oriented immobilization of Abs in the development of test strips has not been elucidated yet.

Typically, in almost all of the LFIA studies, considerably high amounts of pure monoclonal or polyclonal Abs have been used and a little attention has been paid on the possibility of reduction of their consumption. In the present work, we focused on the test zone and we used PG as a host matrix for oriented immobilization of Abs. In addition, we employed the gold enhancement method to boost visual signals. To the best of our knowledge, this is the first report in dual sensitivity enhancement in LFTS for quantification of CEA in real samples with considerably low quantities of Abs.

## 2 MATERIALS AND METHODS

### 2.1 Reagents and instruments

Trisodium citrate (Na_3_C_6_H_5_O_7_·2H_2_O), hydrogen tetrachloroaurate (III) three hydrate (HAuCl_4_·3H_2_O), bovine serum albumin (BSA), Tween‐20, Goat anti‐mouse IgG, and Rabbit anti‐goat IgG (whole molecule)‐FITC were all purchased from Sigma–Aldrich. The CanAg CEA EIA kit was obtained from Fujirebio Diagnostics, Inc., which includes respective antibodies, 3,3′,5,5′‐tetramethylbenzidine (TMB), and standard CEA solutions. The mQ water, produced using Milli‐Q system (>18.2 MΩ/cm), was used for the preparation of solutions. All of the LFTS components were obtained from Nupore Filtration Systems Pvt. Ltd. (India): sample, absorbent, and conjugate pads (SP08, SCL0020215), nitrocellulose membrane (LFNC‐C‐SS03‐15 µm, JCN476015), and Nupore laminates (type‐L).

Surface plasmon resonance absorbance of GNPs and GNPs‐Ab conjugates were recorded using a Cytation 5 Multi‐Mode Reader (BioTek, USA) using a quartz microplate. The field emission scanning electron microscopy (FESEM) images of the strips were captured by FE‐SEM (MIRA3 FEG‐SEM, Tescan, Czech). The size of synthesized GNPs was determined using a transmission electron microscopy (TEM) with 80 kV running voltage (LEO960, Denmark) and the size distribution was calculated using Digimizer free software.

### 2.2 Synthesis of colloidal gold solutions

Turkevich method: a 50 mL aqueous solution of 0.01% HAuCl_4_ was heated to boiling and vigorously stirred; then, 5 mL of 40 mM sodium citrate was added to it quickly. Boiling and stirring were continued for an additional 10 min, thereafter, the solution was cooled down to room temperature with continuous stirring for another 10 min.^21^

Sequential Turkevich method: the half values of the reactants, which were used in the above‐mentioned Turkevich method, were employed for the synthesis of primary gold nanoparticles seeds. Thereafter, the remaining values were used in the second step to grow the primary seeds with a similar procedure.

In both cases, the obtained colloids were passed through a 0.22 µm syringe filter and then centrifuged and re‐suspended in 5 mM borate buffer with pH 7.4, and the optical densities of solutions were set to 1, and then stored in dark bottles at 4°C. All glassware was thoroughly cleaned in aqua regia and rinsed with double distilled water prior to use.

### 2.3 Preparation of GNPs‐Abs conjugated probe

There are two antibodies in the CanAg CEA EIA kit: the Biotin‐tagged anti‐CEA monoclonal antibody (Ab1) and the HRP‐conjugated anti‐CEA monoclonal antibody (Ab2) with concentrations of 3 and 60 µg/mL, respectively. Due to the very low concentration of Ab1, we preferred to immobilize it on GNPs surface and to use the Ab2 as capture antibody on TZ. So, Ab1 was conjugated to the GNPs (OD = 1) through the conventional passive adsorption.^10^ First, 600 µL of Ab1 solution was added drop‐wise to 600 µL of GNPs and the resulting solution was incubated for 60 min shaking at 220 rpm. Then, the GNPs‐Abs conjugates were separated from the solution via centrifugation at 14 000 rpm for 20 min and re‐suspended in the buffer. For blocking the residual sites of GNPs, 300 µL of 5% BSA in borate buffer was added drop by drop, and the stirring was continued for the next 30 min at 220 rpm. Finally, the solution was centrifuged at 14 000 rpm for 20 min, the supernatant was removed, and the pellet of GNPs‐Abs conjugates was re‐suspended in 60 µL of 5 mM borate buffer pH 7.4 containing 10% sucrose. The probe was kept in a dark bottle at 4°C.

### 2.4 Evaluation of conjugation efficiency

After immobilization of Abs on GNPs, the mixture was centrifuged and the supernatant was used in ELISA procedure to evaluate the unbound Abs and hence to calculate the conjugation efficiency. For 15 ng/mL of CEA solution, the same amount of Abs that were used for the preparation of GNPs‐Abs conjugate was evaluated in ELISA procedure as a blank. The CanAg CEA EIA (REF 401‐10) was utilized as an ELISA procedure. The conjugation efficiency (% Conj. Eff.) was calculated as below:

(1) $$\%\;\mathrm{Conj}.\;\mathrm{Eff}.=\left(1-\frac{\mathrm{Ab}s_{620}\;\mathrm{for}\;\mathrm{supernatent}}{\mathrm{Ab}s_{620}\;\mathrm{for}\;\mathrm{blank}}\right)\times 100$$

where Abs_620_ refers to the absorption of solution at 620 nm after the addition of TMB in the ELISA procedure.

### 2.5 Elucidating the role of PG as a host matrix

To clarify the role of PG as a host matrix, studies on microplate surfaces were performed. A 50 µL of 100 µg/mL PG solution was added to each well and left to dry at room temperature. Then, 40 µL of 8 µg/mL goat anti‐mouse IgG was added to each well and incubated for 40 min at 4°C. Thereafter, each well was washed by the running buffer used in LFTS. Then it was blocked by BSA 1% for 20 min. Thereafter, 30 µL of 10 µg/mL FITC‐tagged mouse anti‐goat IgG was added and incubated for 30 min followed by washing three times by the running buffer. Two blank experiments were also carried out by adding just PG and BSA on wells, without adding the PG, with the same other procedures used for the main experiment. The fluorescence intensities of the wells were read by Cytation 5 Multi‐Mode Reader (BioTek, USA) at 528 nm. The data were expressed as mean ± SD. The difference between the groups was obtained by using the Student's *T*‐test. Statistical significance was defined as <0.05. The statistical analyses were performed using GraphPad Prism software version 7.0 (Graph Pad Prism; San Diego, CA, USA).

### 2.6 Preparation of the lateral flow test strips

The pretreatment of pads first carried out as below: the sample pad was dipped into a 5 mM borate buffer pH 7.4 containing 0.05% Tween‐20. The conjugate pad was pretreated by the same solution used for sample pad pretreatment containing 3% sucrose. The pads were then dried at 50°C for 2 h. The pads and NC membrane were subsequently laminated onto the backing card with an overlap between them around to 2 mm. Finally, they were cut into strips with a 3.5 mm width and stored in dry conditions at 4°C for up to 2 weeks. A 2.4 µL of the conjugate probe was pipetted on the conjugate pad for each strip. For the test zone (TZ), 0.2 µL of 100 µg/mL PG solution was first dropped to act as a host matrix for subsequent Abs immobilization. Then, using a micropipette, 0.2 µL of the Ab2 solution was spotted on TZ. For blocking of the remaining sites of PG, different concentrations of BSA solution were examined. For comparison, strips containing just Ab2 or PG at TZ were also prepared and evaluated. For the control zone (CZ), 0.2 µL of PG was pipetted. The whole assembly was then dried at room temperature for 1 h.

For the investigation of the sizes of GNPs on their growth, the above‐mentioned prepared strips were used with just adding PG on TZ as capturing agent and the tests were performed with 0.05% Tween‐20 in borate buffer. The surface of GNPs was blocked and dispersed in the conjugate buffer as described in Section 2.3. The signal amplification solution and image analyzing are the same as described in the following in Section 2.7.

### 2.7 Lateral flow immunoassay procedure

Sample solutions were attained by using standard CEA solutions provided in the kit including 0, 2, 5, 10, 15, 50, and 75 ng/mL. The assay procedure is consisted of firstly dispensing 20 µL of sample solution onto the conjugate pad and keeping for 2 min then adding 80 µL of running buffer to the sample pad. After complete running of the test, for signal amplification, 100 µL of 1:1 (v/v) solutions of 1 mM gold precursor and 2 mM hydroxylamine hydrochloride was freshly mixed and added to the dry strip. Images were captured by mobile phone containing a 16 MP camera and then were analyzed by Image J free software (NIH Image, National Institutes of Health, Bethesda, MD; online at http://rsbweb.nih.gov/ij/). The experiments were carried out in the same light conditions and with one type of smartphone and we did not use the automatic correction option in the camera of mobile phones, to reach more reproducible results.

## 3 RESULTS AND DISCUSSION

### 3.1 Effect of the size of GNPs on their growth

**FIGURE 1:**
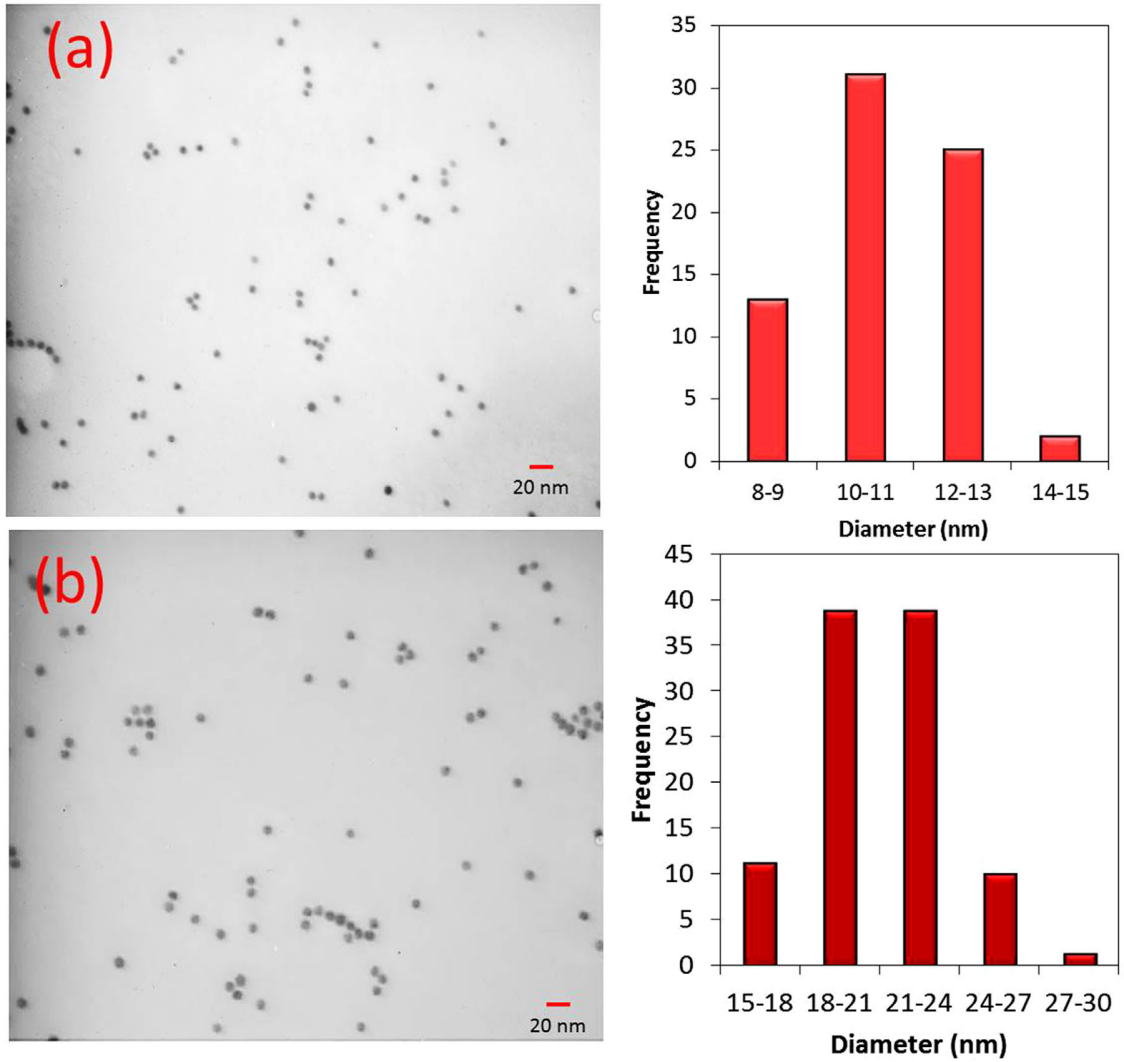
TEM images and size distribution graphs for GNPs synthesized by (a) Turkevich method, (b) sequential Turkevich method

**FIGURE 2:**
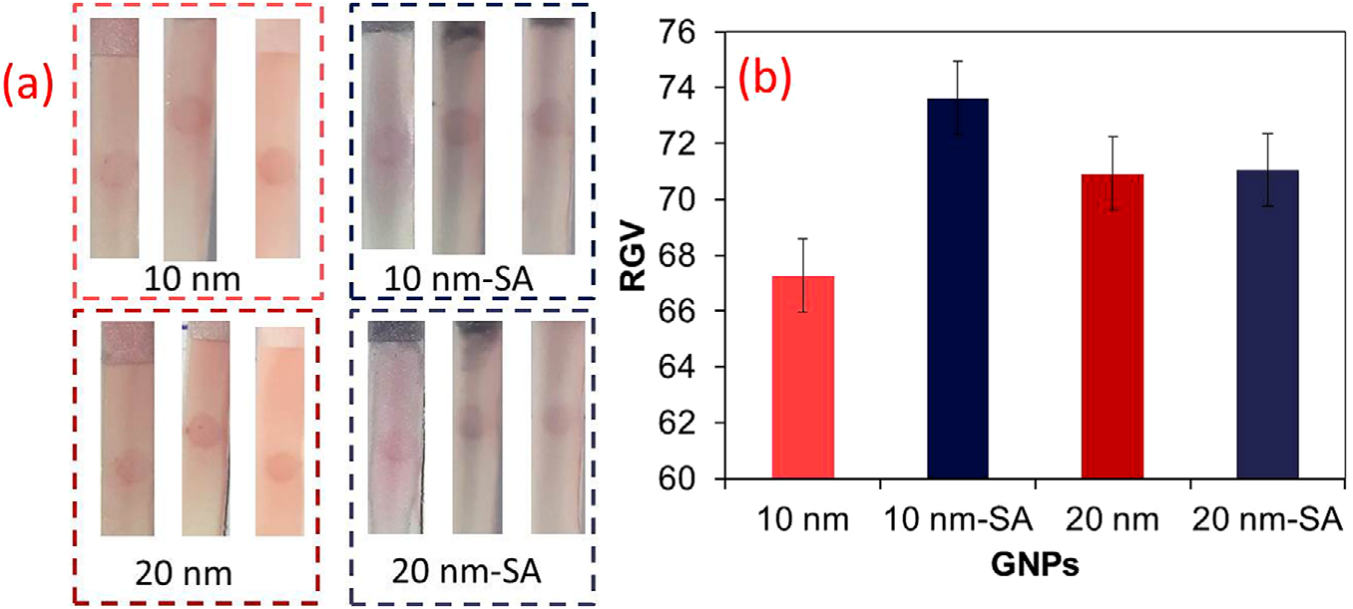
(a) Images of test zone of strips and (b) RGV values for test zone (n = 3, mean±SD), SA represents signal amplification by gold enhancement method

Before the application of the gold enhancement approach in LFIA, the effect of the size of GNPs was elucidated on their growth and hence on signal enhancement. There is a contradiction issue in the gold enhancement approach: on one hand, the large‐sized GNPs benefit from the higher molar absorption coefficient that enables their better visualization and on the other hand, they suffer from low catalytic activity toward gold enhancement compared to smaller sized GNPs. So, we did some experiments to clarify which size of GNPs is better for this purpose. For this means, two dispersions of GNPs were prepared. The first one has an average diameter of 11.40 ± 1.40 nm prepared by the Turkevich method and the second has an average diameter of 20.97 ± 2.49 nm prepared by the sequential Turkevich method. The TEM images and size distribution of GNPs are given in Figure 1. As seen, in both cases, the mono‐dispersed GNPs were attained, which is essential for lateral flow applications. The results of experiments for PG as a capturing agent on the test zone are displayed in Figure 2. The relative gray value (RGV) of TZ, as defined by Equation (2), was used for comparison:

(2) $$\mathrm{RGV}\;=\frac{\mathrm{AG}V_{s}-\mathrm{AG}V_{b}}{\mathrm{AG}V_{b}}\;\times \;100$$

where AGV_S_ and AGV_b_ represent the area of gray value attained by image J for TZ for sample and blank, respectively.

As seen in Figure 2, the large‐sized GNPs provide higher visual signals compared to lower sized GNPs; however, the later shows the better signal amplification ability with more enhanced signals. So, we utilized the GNPs with average diameters of 11 nm for the next experiments.

### 3.2 Evaluating the conjugation of antibodies to GNPs

**FIGURE 3:**
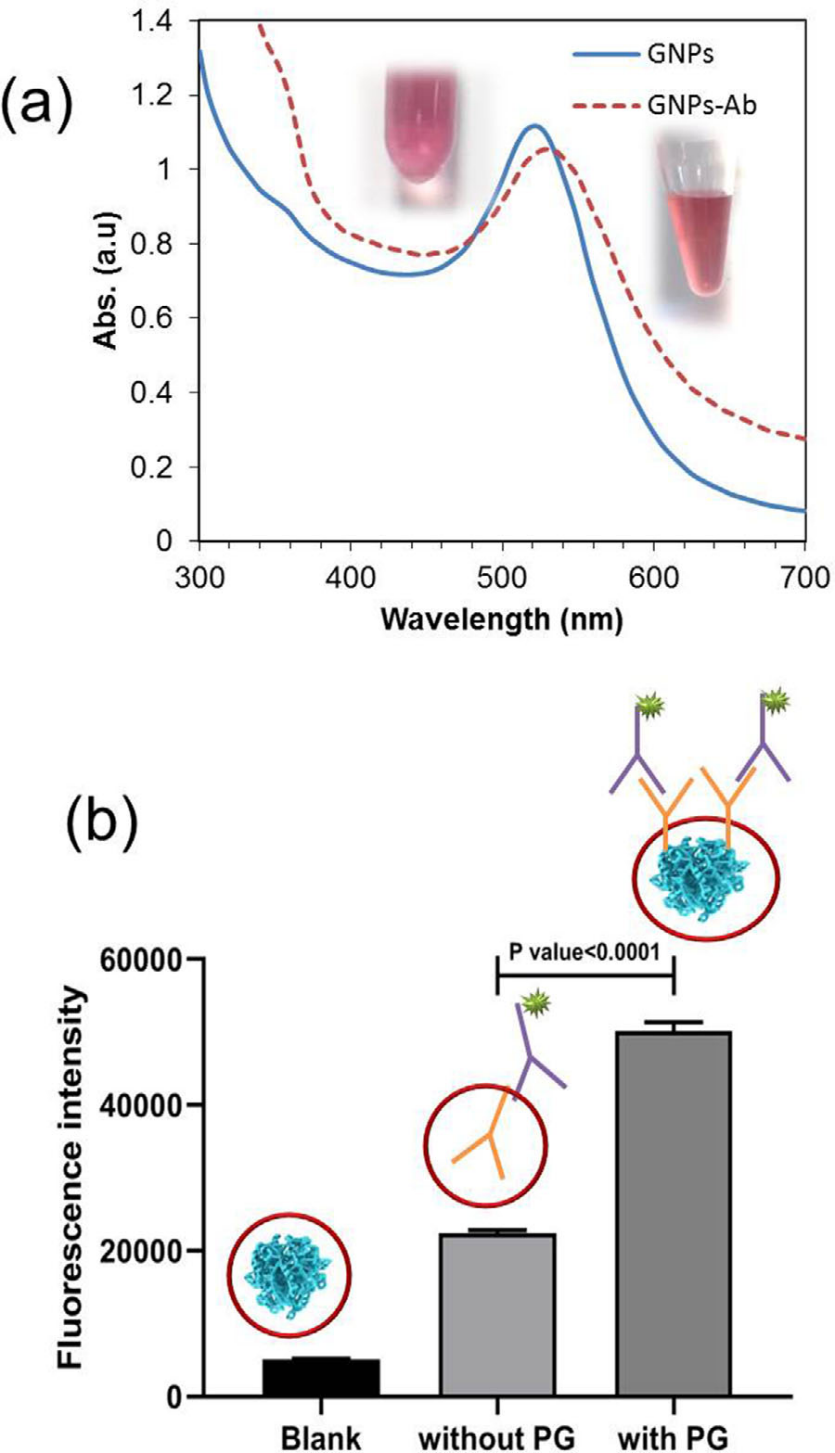
(a) UV‐Vis absorption spectra for GNPs and GNPs‐Ab conjugate (insets show corresponded suspensions), (b) Fluorescence intensities obtained for microplate wells containing Blank (PG and BSA), and goat anti‐mouse IgG attached to rabbit anti‐goat IgG‐FITC in the absence and presence of PG

The UV–Vis absorption spectrum of GNPs revealed the characteristic surface plasmon response (SPR) peak at 520 nm, displaying the nanoparticles has diameters around 20 nm (Figure 3A). After the immobilization step, a red‐shift in SPR was observed showing the possible attachment of Abs on GNPs surface but due to the presence of other components in the antibody solution, other verification experiments were carried out as follows.

Due to the presence of BSA and other reagents in the Abs solution provided in the kit, the red‐shift in SPR peak (Figure 3A) could not be exactly attributed to the formation of GNPs‐Ab conjugate. Hence, we developed an ELISA‐based approach for quantification of immobilized Abs on GNPs surface. After incubating the Ab1 solution with GNPs, the mixture was centrifuged and the supernatant was evaluated in ELISA to obtain the amount of un‐attached Abs. The values of Abs_620_ for blank Ab1 solution and the supernatant were obtained as 0.36 ± 0.02 and 0.21 ± 0.04, respectively. Thus, a *%Conj. Eff*. of about 41% was achieved, which represents the immobilization of 0.738 µg of mAb per 600 µL of GNPs. In theory, considering a 45 nm^2^ for each antibody surface and an average diameter of 11 nm for GNPs, the maximum number of antibodies per each particle is 8. On the other hand, calculating the number of GNPs and Abs in each preparation batch shows that the ratio of Ab:GNP is 3.25:1. In practice, the %*Conj. Eff* value represents that the number of Abs per each GNP is about 1.3 (see Supporting Information for calculations). Since the Abs could form strong S‐Au bonds with GNPs via disulfide bonds at the hinge region of their structure, so their attachment to GNPs is preferred to electrostatic attractions, which may involve by other available components in the solution.

### 3.3 Elucidation the formation of sandwich structures on the plate and within NC membrane

Since the %*Conj. Eff*. just presents the loading of Abs on GNPs, to evaluate the efficiency of the probe in the formation of sandwich structures, the prepared GNPs‐Ab1 probe was used in the ELISA process. A value of 0.12 ± 0.03 was obtained for Abs_620_ (compared to 0.36 ± 0.02 for neat Abs), representing the formation of sandwich structure on the plate and the good efficiency of the probe. After ensuring good performance of the designed probe in homogenous ELISA, the formation of the sandwich structure on the TZ without the PG as a host matrix was checked. In the absence of PG, the assay failed to show a considerable visual signal. This is maybe due to the low concentration of Ab2 in the test zone and its washing during the running of assay and due to its random orientation within the membrane. However, by the introduction of PG, it may maintain the capture Ab2 and further enhances the oriented immobilization of Ab2 through interaction by the FC region of Ab2. To verify the effect of PG, first, studies on microplate was performed as described in Section 2.5. As seen in Figure 3b, considerably higher fluorescence intensity was obtained for the microplates containing PG as a host matrix (5097 ± 62, 22397 ± 425, 50152 ± 1197 for blank, without PG, and with PG, respectively). The blank fluorescence is attributed to the attached BSA and PG on wells. On the other hand, since the main interaction of PG with Abs is hydrophobic so it can be concluded that the Fab regions of Abs are free for attachment to secondary Ab. The *P*‐value < .0001 shows the difference between the two groups is statistically significant. This difference is attributed to the role of PG as a host matrix for maintaining and oriented immobilization of Abs.

**SCHEME 1:**
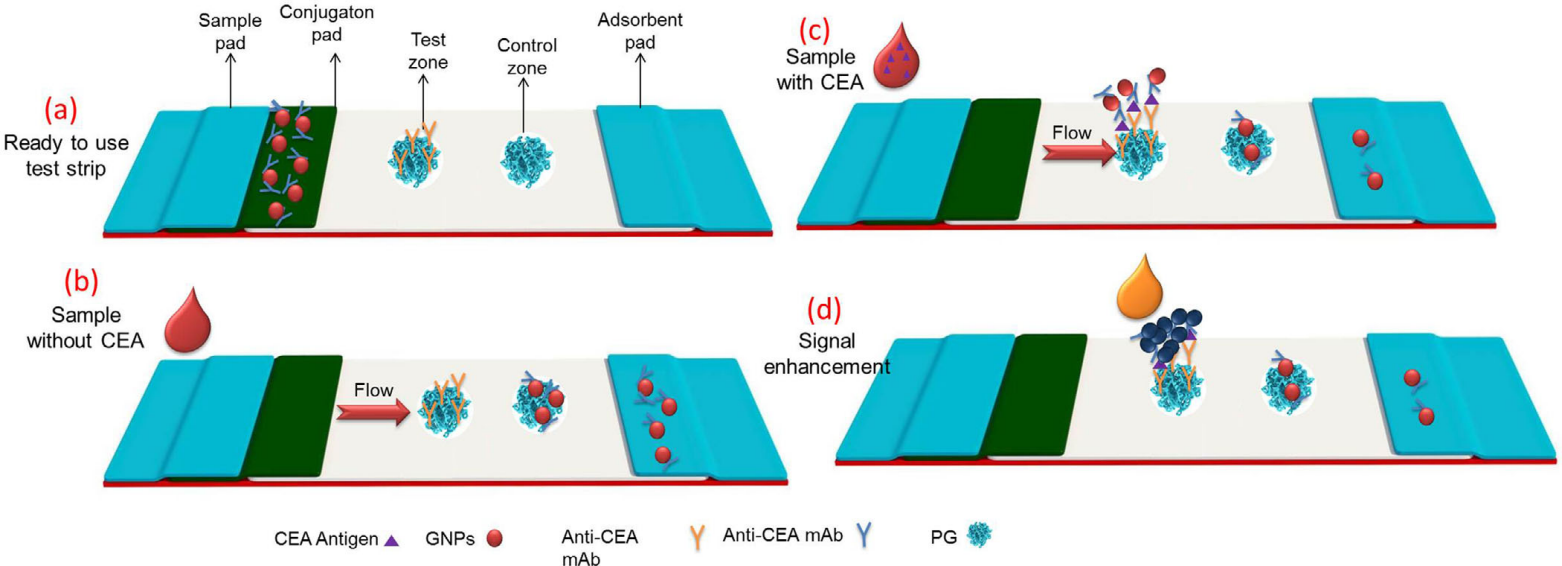
Schematic representation of (a) ready to use lateral flow test strip and its components and the role of PG for oriented immobilization of capture antibodies, running the test strip in the absence (b) and in the presence (c) of CEA, (d) signal enhancement by the addition of gold precursor and hydroxylamine hydrochloride

**FIGURE 4:**
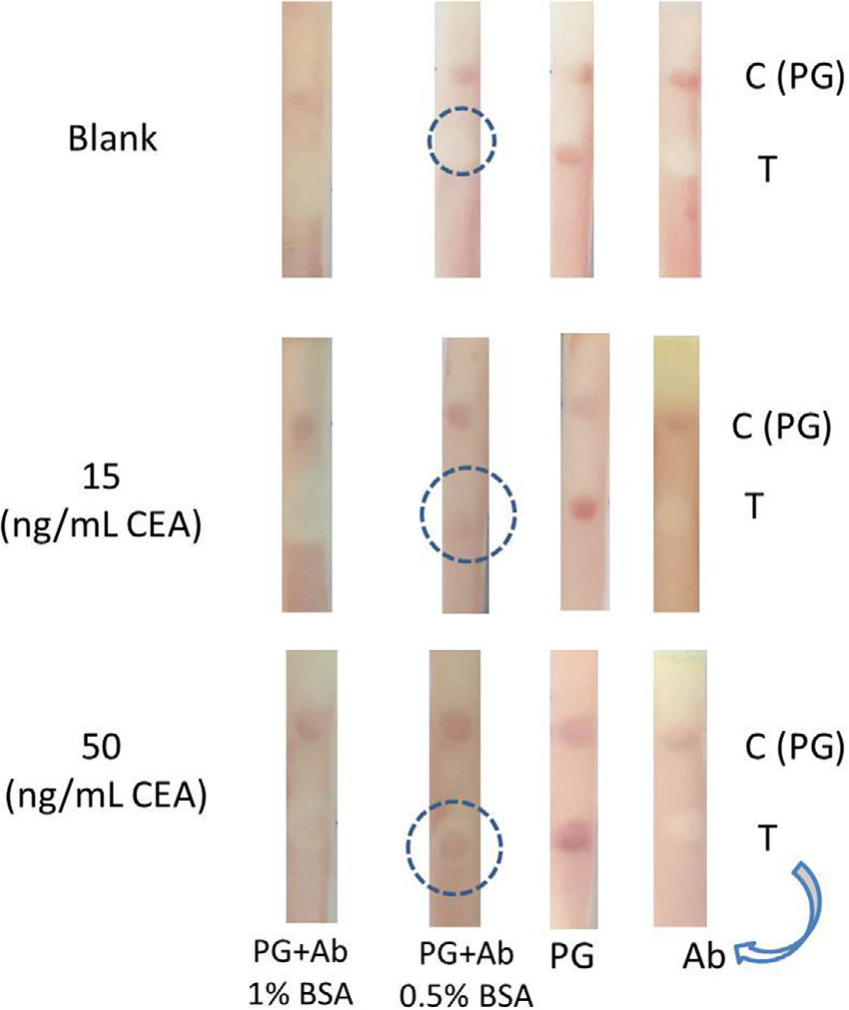
Lateral flow test strips representing the results obtained for different concentrations of CEA, 0 (Blank), 15, and 50 ng/mL when Ab, PG, and both of them were dropped on test zone and blocked by 0.5% and 1% BSA

A schematic of the assay is shown in Scheme 1, which displays the components of the strip, the role of PG as a host matrix for oriented immobilization of capture antibodies (a), and the results for running the assay in the absence (b) and presence of the target (c) followed by the gold enhancement step (d). Since the PG acts as a capture agent for GNPs‐Ab too, thus it was used in the CZ to reduce further the Abs consumption. However, it is necessary to block the TZ after immobilizing the Abs. So, different concentrations of BSA including 0%, 0.5%, and 1% was checked for blocking. By comparing different conditions of the TZ, as seen in Figure 4, for strips containing PG and capture Abs blocked by 0.5% BSA, a considerable change in color was observed for different concentrations of CEA (represented by dashed circles) with less background, confirming the role of PG as an efficient immobilization matrix.

### 3.4 Optimization of assay conditions

In this work, the amount of Ab2 immobilized on the test zone, the constituents of running buffer, and the mixture of signal enhancement reagents was optimized to reach an enhanced visual signal. Different concentration of Ab2 was applied on TZ to check its amount on assay performance. The concentration of Ab2 within the kit is 60 µg/mL; then by its pipetting on TZ for first time, second time, and third times, various amounts of it were evaluated. For several times dropping, it caused the aggregation of GNPs and impeded the lateral flow mainly due to the presence of a high concentration of salts in Abs solution; hence, the experiments were carried out by 60 µg/mL of Ab2.

The running buffer has a considerable effect on flow rate and thus on assay time and its performance. Two buffers including PBS and borate each with 0.05%, 0.1%, and 0.5% of Tween‐20 were investigated as running buffers on assay performance. The assay could not work properly by PBS and the higher concentration of surfactant in borate buffer washed the attached structures on TZ without remaining any visual signal.^22^ Thus, the borate buffer with 0.05% Tween‐20 was selected as the optimum running buffer. The ratio of two components of signal amplification reagents was also investigated to obtain an amplified visual signal with less background. Under the experimental conditions, the 1:1 ratio of 1 mM Au^3+^ and 2 mM hydroxylamine hydrochloride showed the desired characteristics and used for the following experiments (Data are not shown here). At the optimized conditions, the assay time is below 10 min.

### 3.5 Analytical utility of developed test strip

**FIGURE 5:**
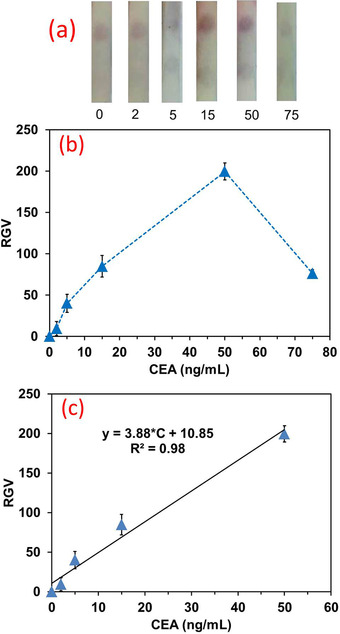
(a) Photographs representing the strips and test zones, and (b) the corresponding RGV vs. standard solutions of CEA, (c) Calibration graph within the range of 0 to 50 ng/mL

In optimal conditions, the detection of target was performed by adding standard values of CEA on the conjugation pad, followed by the addition of running buffer to the sample pad. By employing borate buffer with 0.05% Tween‐20, the strip worked well and a linear correlation between CEA concentration and the relative gray value (RGV) of TZ, as defined by Equation (1), was used for quantification. The results showed a linear range of 2‐50 ng/mL with an *R* ^2^ value of 0.98 and a LOD value of 0.35 ng/mL (3σ/m) (Figure 5). For the higher concentrations of CEA, the visual intensity was reduced possibly due to the hook effect (concentration of 75 ng/mL in Figure 5A,B), which arises from the occupation of capture sites of Abs in high concentrations of target molecules. The presence of hook effect is the main drawback of immunoassays and it has been observed both in LFIA and ELISA. This phenomenon is observed when the user is expected to see high amounts of the target but suddenly faced with low amounts of it. In this situation, the main solution which dominantly is recommended in commercial products is the dilution of the samples and checking again via the strip. If the signal intensity is enhanced by dilution, the hook effect is likely to occur.^23^, ^24^

### 3.6 Serum samples analysis

**FIGURE 6:**
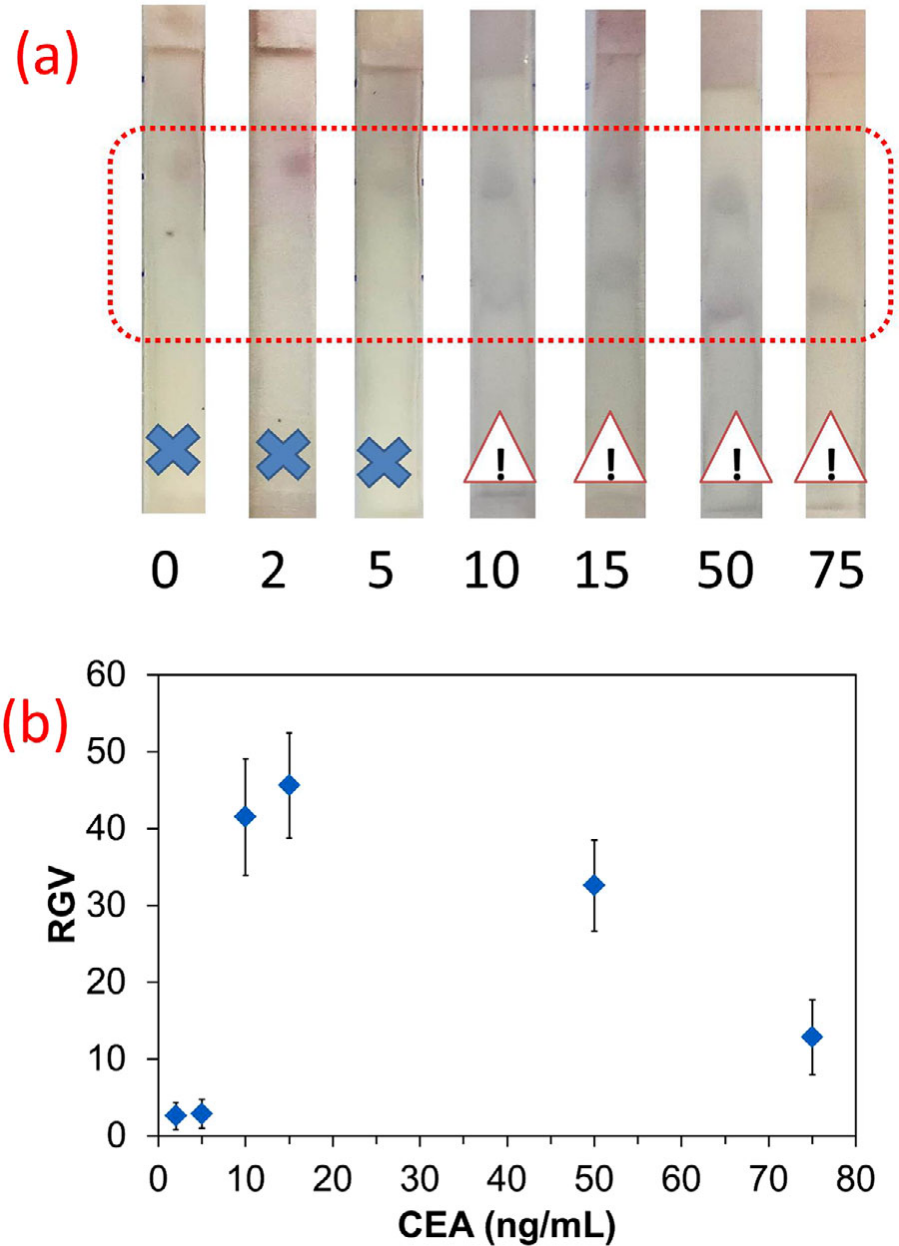
Photographs of strips after running with serum samples, showing a dark blue color in the test zone for concentrations higher than 10 ng/mL of CEA, (b) corresponding RGV vs. CEA (ng/mL) graph

To evaluate the applicability of developed strip for real sample analysis, standard solutions of CEA were added to serum samples and then evaluated in the strip. Due to the difference in the matrix, the employed running solution used for the analysis of CEA in the buffer could not run the strips, so the percentage of surfactant was increased up to 0.1%, which enabled the successful running of the assay within 15 min. The strip could successfully run with the serum samples (Figure 6A) but the trials for quantitative measurements were failed and the study was limited to qualitative analysis with a cut‐off value of 10 ng/mL (Figure 6B). This value is in the range of clinical importance for the analysis of CEA in real samples.^4^, ^25^ A comparison between the results attained for standard CEA solutions and the serum samples show that the controlled growth of GNPs should be carried out to reach quantitative results. So, the nature of matrix not only affects assay time but also impresses on the growth of GNPs and hence on the assay performance.

**FIGURE 7:**
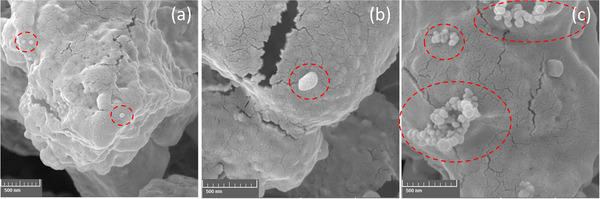
FESEM images for test zone (a) without gold enhancement, and with gold enhancement in the buffer (b) and serum sample analysis (c) (the discussed structures are shown by dashed circles)

To further elucidate the assay and to clear the attained results, the FESEM images of TZ after running the test, and also after signal amplification are shown in Figure 7. These images clearly show the attachment of GNPs within the porous environment of NC at TZ (Figure 7A). Furthermore, the enhancement solution increased the size of GNPs effectively up to the submicron range (Figure 7B). In addition, the morphologies of the enlarged GNPs in serum analysis are different from those employed for buffer solution and show many submicron‐sized GNPs attached to each other (Figure 7C). The formation of such structures is probably the main reason for the inability to quantify the serum results. This is maybe due to the presence of BSA and other constituents in serum that affects the wicking rate and thus reaction time, and hence on the resultant morphology and color intensity.^26^

A comparison was also made between some characteristics of our designed strip and the reported literature in detection of CEA in Table 1. The amount of used capture antibody was calculated as (dispensing rate × width of strip × concentration of antibody solution) or (volume of antibody × concentration of antibody solution). In the case of works that did not report the dispensing rate, a usual value of 1 µL/cm was used for calculation. Although the dual sensitivity enhancement strategy just provided qualitative results for real samples analysis and its sensitivity is several orders of magnitude lower than compared with some of the previously developed lateral flow immunoassay systems based on other principles such as the work reported by Liu et al.,^27^ however, it enabled a considerable reduction in Abs consumption with a simple signal boosting procedure, the achievement that is highly important from both practical and economic viewpoints. The weak signals in our work are mainly due to two reasons: the first one is the application of low amounts of antibodies in test strips that result in weak but detectable signals, and the second reason is the employment of a drop‐based approach for creating test and control zones. So, the results may be improved by employing high quantities of antibodies and utilizing suitable dispensers for the creation of test and control zones. Due to its some desired characteristics such as simplicity, low detection time, and less cost, the developed strategy is desired for point‐of‐care applications in resource‐limited settings.

**TABLE 1:** Comparison of dual sensitivity enhancement lateral flow immunoassay with some of the previous works

Detection approach	Abs in test zone (µg)	Abs in control zone (µg)	Reporting label	Signal amplification	Linear range (ng/mL)	Detection limit (ng/mL)	Assay time (min)	Reference
Fluorescent	NR	NR	Quantum dots	–	1‐100	5	20	^28^
Fluorescent	0.6	0.3	Quantum dot beads	Quantum dot beads	1‐50	0.0378	15	^29^
Fluorescent	0.48	0.36	quantum dot‐doped polystyrene nanoparticles	–	2.8‐680	0.35	15	^30^
Magnetic	0.6	0.3	Iron oxide nanoparticles	–	1‐100	0.045	30	^31^
Image analysis	NR	NR	Iron oxide nanoparticles containing Abs and biotin tagged oligonucleotides	Streptavidin‐biotin interaction	0.25‐1000	0.25	15	^27^
Naked‐eye detection	0.012	0	GNPs	Oriented immobilization of Abs and gold enhancement	2‐50	0.35	15	In this work

NR, not reported.

## 4 CONCLUSIONS

In summary, the oriented immobilization of capture Abs and gold enhancement were utilized to develop dual sensitivity enhancement in LFTS with a considerable reduction in the consumption of antibodies. Although the results showed a considerable decrease in the activities of Abs in porous NC membrane compared to homogenous conditions, benefiting from the gold enhancement method and oriented immobilization of antibodies via protein G as a host matrix, the developed LFTS enabled quantification of low levels of CEA in buffer samples with considerably low quantities of Abs. In addition, the developed LFTS provided a qualitative assay for point‐of‐care applications with a cut‐off value of 10 ng/mL. The nanoparticle‐based LFIA benefits from the simple operation, less sample consumption, and more user‐friendly characteristics compared to the previously reported immune‐dipstick assays. Besides the desired characteristics of our developed strategy, utilizing standard monoclonal antibodies may further improve the attained results and the efficiency of the developed strip. Also, the development of smartphone‐based *apps* and imaging systems to remedy the undesired effects of smartphone‐based imaging and also the effects of environmental light conditions are interesting for obtaining better results.

## CONFLICT OF INTEREST

There are no conflict of interest associated with this work.

## AUTHOR CONTRIBUTIONS

T. Mahmoudi and B. Shirdel performed the research. T. Mahmoudi and B. Baradaran designed the research study. B. Baradaran contributed essential reagents or tools. T. Mahmoudi, B. Baradaran, and B. Mansoori analyzed the data. T. Mahmoudi and B. Baradaran wrote the paper.

## Supporting information

Supporting Information
