# Supplementary material for: Dual sensitivity enhancement in gold nanoparticle‐based lateral flow immunoassay for visual detection of carcinoembryonic antigen
Source: Anal Sci Adv. 2020 Jun 23;1(3):161–72. doi: 10.1002/ansa.202000023 (PMC10989072; doi:10.1002/ansa.202000023)
Supplement: Supplementary file 1 — Supporting Information [file ANSA-1-161-s001.docx]

**Electronic Supplementary Material for “Dual Sensitivity Enhancement in Gold Nanoparticles-based Lateral Flow Immunoassay for Visual Detection of Carcinoembryonic Antigen”**

Tohid Mahmoudi^1^, Behnaz Shirdel^2,1^, Behzad Mansoori^1,3^, Behzad Baradaran^1*^

^1^ Immunology Research Center, Tabriz University of Medical Sciences, Tabriz-Iran

^2^ Student Research Committee, Tabriz University of Medical Sciences, Tabriz, Iran

^3^ Department of Cancer and Inﬂammation Research, Institute of Molecular Medicine, University of Southern Denmark, Odense, Denmark

Corresponding authors at:

Immunology Research Center, Tabriz University of Medical Sciences, Tabriz, Iran

E-mail address:

baradaranb@tbzmed.ac.ir (B.Baradaran)

Tel: +98 41 3337 1440; Fax: +9841 33371311

1. **The calculation of the number of attached antibodies per each gold nanoparticle (GNP):**

The calculated surface area for each spherical GNP with a diameter of 11 nm is 380 nm^2^ and the approximate surface area for each mAb is 45 nm^2^. In theory, the maximum number of Abs per each GNP will be 380/45 ≈ 8.5.

The number of gold atoms per each GNP is calculated using eq. (1):

$N=\frac{\pi}{6}\frac{\rho D^{3}}{M}=30.89602 D^{3}$ eq. (1)

where *ρ* is the density for fcc gold (19.3 g/cm^3^) and M stands for the atomic weight of gold (197 g/mol). For D = 11 nm, N = 41122.

The molar concentration of the nanosphere solutions was calculated by dividing the total number of gold atoms (N_total_, equivalent to the initial amount of gold salt added to the reaction solution as described in section 2.2) over the average number of gold atoms per nanosphere (N) according to eq. (2), where V is the volume of the reaction solution in liter and N_A_ is the Avogadro’s constant. Since the quantity of citrate is much higher than that of gold precursor (200 μmol *vs.* 12.69 μmol), It is assumed that the reduction from gold(III) to gold atoms was 100% complete.

$C=\frac{N_{total}}{NVN_{A}}$= 0.0061719 μM eq. (2)

In 600 μL of GNPs suspension, the number of GNPs will be 2.229 × 10^12^. Considering the molecular weight of Ab as 150 kDa, 600 μL of mAb (3 μg/mL) contains 7.224 * 10^12^ Antibody molecules. So, in each batch a mAb: GNP ratio of 3.2 is obtained.

In addition, considering a *%Conj. Eff* of %41, the number of Abs attached to GNPs is 2.962 * 10^12^. So an Abs: GNPs ratio of 1.3 is achieved, which is just 15% of theoretical calculations.

**References:**

[1] Liu, X., Atwater, M., Wang, J., & Huo, Q. (2007). Extinction coefficient of gold nanoparticles with different sizes and different capping ligands. Colloids and Surfaces B: Biointerfaces, 58(1), 3-7.‏
